# A Multimodal Localization Framework Design for IoT Applications

**DOI:** 10.3390/s20164622

**Published:** 2020-08-17

**Authors:** Michiel Aernouts, Filip Lemic, Bart Moons, Jeroen Famaey, Jeroen Hoebeke, Maarten Weyn, Rafael Berkvens

**Affiliations:** 1 IDLab—Faculty of Applied Engineering, University of Antwerp—imec, 2000 Antwerp, Belgium; maarten.weyn@uantwerpen.be (M.W.); rafael.berkvens@uantwerpen.be (R.B.); 2 IDLab—Department of Computer Science, University of Antwerp—imec, 2000 Antwerp, Belgium; filip.lemic@uantwerpen.be (F.L.); jeroen.famaey@uantwerpen.be (J.F.); 3 IDLab—Department of Information Technology, University of Ghent—imec, 9000 Ghent, Belgium; bamoons.moons@ugent.be (B.M.); jeroen.hoebeke@ugent.be (J.H.)

**Keywords:** IoT, multi-RAT, multimodal, localization, LPWAN

## Abstract

Multiple Radio Access Technology (multi-RAT) communication with Low Power Wide Area Networks (LPWAN) significantly increases the flexibility of Internet of Things (IoT) applications. Location-based services that build upon such a multimodal communication architecture are able to switch to an optimal localization method depending on the constraints of the active wireless technology. Furthermore, the resulting location estimate can aid location-based handover mechanisms to reduce the energy consumption of a multi-RAT IoT device. In this research, we present our design of a multimodal localization framework and illustrate the benefit of such a framework with two IoT use case examples. For the first use case, valuable artwork is tracked during transportation to a museum. In the second use case, we monitor the usage and location of large construction tools. Finally, we propose how our localization framework can be improved to deal with implementation challenges and to reduce location estimation errors.

## 1. Introduction

Internet of Things (IoT) devices rely on wireless communication protocols to transmit and receive application data. For this purpose, network operators have been deploying Low Power Wide Area Networks (LPWANs) such as LoRaWAN and Sigfox on a large scale [1]. Via these sub-gigahertz (sub-GHz) networks, IoT devices are able to exchange data over multiple kilometers at low throughput, while maintaining a low power consumption. In order to reduce the hardware cost and complexity, a single wireless communication module is integrated in a device. Unfortunately, this also reduces reliability and flexibility, which limits the device’s suitability for IoT applications. In order to regain flexibility without losing the benefits of simple hardware design, we have been working on multiple Radio Access Technology (multi-RAT) communication for IoT devices [2].

Apart from exchanging application information, LPWAN communication is also used to locate a transmitting IoT device. Unlike Global Navigation Satellite System (GNSS) solutions such as Global Positioning System (GPS) and Galileo, LPWAN localization does not require additional battery-draining hardware to obtain a location estimate. Moreover, LPWAN enables outdoor as well as indoor localization which is an important requirement for many IoT use cases. However, the estimation error of these localization methods can range from tens of meters up to hundreds of meters depending on the constraints of the available network. Consequently, switching between networks via multimodal communication can result in reduced location estimation errors, which is beneficial for IoT devices that operate in multiple environments. For example, an error of hundreds of meters might be sufficient for asset tracking during transport. On the other hand, indoor localization for warehouse management requires a higher location accuracy. This requirement can be met if an IoT tracking device is able to switch to a more optimal network upon arrival.

Whereas related work on multi-RAT focuses on communication reliability and energy efficiency of IoT devices, we investigate the benefits of multi-RAT for location-based services. Rather than reporting the estimation errors of specific localization methods for LPWAN, this paper presents our novel concept of a multimodal localization framework for IoT applications. The purpose of our framework is to estimate the location of a multi-RAT IoT transmitter in the most optimal way, depending on the constraints of the LPWAN that is actively used. Additionally, the framework can be used in conjunction with a Virtual Network Operator (VNO) that is responsible for device management, data exchange, and location-based handover algorithms. The frameworks’ resulting location estimate can assist such an algorithm in its decision to switch between RATs.

To illustrate the benefits of our concept, we describe the expected outcomes of implementing the framework in two IoT use case examples:

- An artwork tracking application for museums.
- A track-and-trace application for construction equipment.

The remainder of this paper is structured as follows. In Section 2, the related work on multi-RAT communication and localization in LPWAN is described. Section 3 explains the workflow of our multimodal localization framework, as well as the related communication architecture that takes care of the handover between networks. In addition, we consider how the reliability of our localization methods can be estimated. Section 4 demonstrates the expected location accuracy that can be achieved when implementing our framework in two IoT use cases, based on the results of related LPWAN localization research. Finally, Section 5 concludes the paper and lists our future work.

## 2. Related Work

In this section, we discuss the related work regarding multi-RAT communication and localization. Because our experiments were conducted with Sigfox, LoRaWAN and DASH7, we provide an overview of the location accuracy that can be achieved with these technologies in different environments. The expected location accuracy of the use cases in Section 4 are based on results from this related research.

### 2.1. Multi-RAT Communication and Localization

The growth of the IoT has caused a trend towards multi-RAT IoT devices, i.e., a single device supporting multiple modulation schemes and communication technologies. Such devices are able to switch at run-time between different RATs and can therefore support long range, low data rate technologies combined with medium range, higher data rate technologies.

Mikhaylov et al. experimented with a multi-RAT IoT device that transmits packets through a public Narrowband-IoT (NB-IoT) network and a private LoRaWAN network. With this device, they demonstrate that multi-RAT LPWAN enables more reliable communication because of the ubiquitous network coverage and decreased packet loss [3]. Furthermore, they established that multi-RAT can improve the energy efficiency of IoT devices [4].

The benefits of multi-RAT for location-based services are also being recognized by the research community. In [4], researchers developed a device that includes Bluetooth Low Energy (BLE), LoRaWAN, NB-IoT, and GNSS technology to investigate the feasibility and benefits of multi-RAT IoT devices for localization technologies. With this device, they demonstrate that multi-RAT can enable energy consumption and balances the achievable location accuracy. For example, an indoor BLE network is able to report a location estimate with an error of only 1 m, whereas an error of more than 500 m was achieved in a large outdoor LoRaWAN network. However, adding all these different technologies on a single device requires a variety of hardware that increases the cost of the device. In our research, we experiment with a single-chip multi-RAT device that can switch between Sigfox, LoRaWAN, and DASH7. With these technologies, we provide a dynamic localization solution for indoor and outdoor environments. Furthermore, the research in [4] mainly focuses on the energy consumption of their multi-RAT device, whereas our research explores the implementation of a backend architecture that supports multi-RAT devices in hand-over mechanisms and location-based services.

In [5], both Wi-Fi and Bluetooth measurements are used for fingerprinting localization in airports, resulting in estimation errors of 2 m to 15 m. Although these results are very suitable for many indoor localization applications, this multimodal approach cannot be extended to large outdoor environments. Contrary to [5], our multi-RAT device can still be located in large outdoor environments by switching to a public LPWAN.

Rodas et al. also acknowledge the benefit of multi-RAT for localization [6]. In their research, they propose an architecture that combines data from multiple RATs for localization purposes. However, this architecture mainly focuses on indoor localization and does not consider the possibility to assist multi-RAT devices with location-based handover mechanisms. In our work, we demonstrate a localization framework that cooperates with such a mechanism via two IoT examples that include both indoor and outdoor localization.

### 2.2. LPWAN Localization

To illustrate the location accuracy that can be obtained through localization with LPWANs, this section presents some of the research community’s contributions on the topic. Generally, wireless localization methods can be categorized into three sub-categories.

First, a transmitter can be located with the Received Signal Strength (RSS) at the receiving LPWAN gateways. Anagnostopoulos et al. applied *k*NN fingerprinting on a large dataset with Sigfox messages that were collected in an outdoor urban area [7]. By optimizing hyperparameter *k*, signal space distance metrics and RSS data representation, they were able to achieve a mean error of 319 m. In [8], Sigfox fingerprinting was compared with other RSS-based methods, namely proximity and ranging with varying RSS propagation models. This comparison demonstrated that fingerprinting in outdoor environments yields smaller estimation errors than other methods. However, building and maintaining a fingerprinting database has proven to be an arduous task, so, despite their higher errors, other RSS-based methods which are easier to implement can still be advantageous. Although it is not considered to be an LPWAN due to its medium range, DASH7 has some benefits that make it a relevant technology that should not be overlooked when designing a multimodal communication architecture for IoT applications. Like LoRaWAN and Sigfox, DASH7 operates in the 868 MHz frequency in Europe, allowing a single-antenna device to switch smoothly between those networks. In [9], Berkvens et al. evaluated indoor localization with DASH7 using a multi-wall propagation model and found that this approach leads to a mean error of 1.27 m. Hence, it is interesting to implement this method in a multimodal localization framework for scenarios that require a high location accuracy, e.g., asset tracking inside a warehouse.

Second, localization can be performed using timing information. For IoT applications, Time Difference of Arrival (TDoA) methods are more suitable than Time of Arrival (ToA) methods because TDoA synchronization between the transmitter and the gateways is not required, only the gateways need to be synchronized to each other. However, the receiving gateways have to detect the arrival time of a signal very precisely to limit the magnitude of location estimation errors. Therefore, time-based localization is not applied to Sigfox due to its Ultra Narrowband (UNB) characteristic [10]. On the other hand, TDoA has been adopted for LPWAN standards with a larger bandwidth, e.g., LoRaWAN. Researchers achieved a median error of 200 m using the raw TDoA data [11]. After taking map information and sensor data into account, the median error decreased to 75 m. Fargas et al. demonstrated TDoA localization for static LoRaWAN transmitters, in a network with four gateways. After averaging the measurements, a mean accuracy of approximately 100 m was attained [12]. In general, time-based localization with LPWAN produces more accurate location estimations than RSS-based methods. This is an important observation that can be taken into account when a localization framework has to decide upon an optimal algorithm.

Third, the Angle of Arrival (AoA) of a wireless signal can be used to calculate a location estimate. Typically, AoA employs costly and complex receiver hardware that is able to detect from which direction a wireless signal is received. With two AoA-enabled gateways, the location of a transmitter can be computed. Of course, the estimation accuracy strongly depends on the distance between the transmitter and the gateways, as a small inaccuracy in the angle estimation leads increasingly large estimation errors if the transmitter is further away. To the best of our knowledge, AoA has not been evaluated with LPWANs in a realistic scenario. However, BniLam et al. introduced a low-cost sub-GHz AoA gateway which could be used to implement AoA for LoRaWAN [13]. This gateway was recently used to combine TDoA and AoA localization in an outdoor LoRaWAN network that covers a dense urban area [14]. In this previous research, a mean error of 122 m is obtained by using a particle filter to combine TDoA and AoA.

The goal of this paper is not to improve the performance of these individual localization algorithms. Instead, we propose a framework that combines multiple localization approaches to enable localization for multi-RAT IoT devices in multiple environments. In our use case examples of Section 4, we use the results of the aforementioned related work as a guideline to illustrate the expected estimation accuracy of our localization framework.

## 3. Multimodal Localization Framework

In this section, we describe the workflow of our multimodal localization framework. As the localization algorithms strongly depend on the existing communication link, it is important to first explain how multimodality can be achieved in the communication layer. Therefore, the first subsection introduces our work on a multi-RAT communication architecture that enables location-based discovery and vertical handovers in outdoor LPWAN communication. In the second subsection, we clarify the design of our localization framework and how it can be implemented on top of the multi-RAT communication architecture. Finally, the third subsection describes our work on estimating the reliability of a location estimate, which we intend to add to our localization framework in our future work.

### 3.1. Multi-RAT Communication Architecture

Different sub-GHz LPWAN technologies select their communication ranges based on available data-rates and energy consumption. To increase the flexibility and reliability of communication, a technology with a very long range could be used for basic connectivity, while another one could simultaneously be used intermittently for data-offloading purposes. This has been recognized in the community and several multi-RAT devices have been proposed [2,15]. The usual procedures for the discovery of or handover between LPWAN technologies are based on either continuous beaconing, continuous probing, or blind data transmission. In multi-RAT scenarios, such methods can cause high energy dissipation and signaling overhead, which is principally undesired for sub-GHz LPWAN technologies that are targeting low-power performance and have duty-cycle constraints.

To address this issue, we have proposed a location-based and location-quality-aware mechanism for the discovery of and vertical handover between sub-GHz LPWAN technologies in outdoor environments. As many of the IoT use-cases supported by LPWAN technologies require localization capability of a mobile device, the location information can also be utilized for optimizing different aspects of LPWAN communication, such as the discovery and handover procedures. However, as the location estimates in practice generally feature certain levels of localization errors, these imperfections should be accounted for in any location-based optimization of wireless networks. Hence, the proposed mechanism, in addition to the location information of the mobile device and a receiver, in its operation explicitly accounts for the localization errors in the location information of the mobile device. The discovery and handover decisions are then based on the expected Signal-to-Noise Ratio (SNR) between the transmitter and the receiver, which are obtained from their location information, error estimates, and propagation characteristics of the deployment environment. The mechanism significantly reduces the overhead of probing, listening for beacons, or blind data transmissions, as they can now be performed only when there is a considerable chance of a desired LPWAN technology being available.

Using Sigfox and LoRa technologies, we have experimentally evaluated the accuracy of the mechanism in making discovery and handover decisions. We have demonstrated its high accuracy for the GPS-accurate location information of the mobile device. We have also shown that our approach can be successfully utilized for the discovery of LPWAN technologies even for an order of magnitude less accurate location information of the mobile device. More details on the proposed mechanism and the achieved results can be found in [16].

However, as the performance decreases with a decrease in localization accuracy, resulting in too conservative positive discovery decisions and thus requiring a GPS for a practical implementation, we implemented a more energy-efficient mechanism. This vertical handover algorithm uses periodic polling for the available technologies in order to fall back to the best one available. Nevertheless, by providing signaling slots, information from our multimodal localization framework or the location-quality-aware mechanism can be used to update the algorithm parameters in order to make it more resilient and energy efficient. Section 4.2 illustrates its usage on the basis of a use case. A more in-depth theoretical disquisition and simulation based evaluation can be found in [17].

In order to deal with the increased complexity of multiple RATS on a single device and to enable our multi-RAT handover algorithms, a novel architectural approach is required. In our previous work, we proposed the concept of a modular, cloud-based VNO as a solution to deal with multi-RAT IoT networks [15]. For every communication technology the device will make use of, the VNO should be able to interface with the network infrastructure. Data received from the LPWAN should be forwarded to applications consuming it and data coming from the applications should be delivered to the correct LPWAN. For each technology, a different data delivery approach is required. Messages for a LoRaWAN class A device, for example, should be queued until the next uplink transmission, since downlink communication is only available after an uplink. DASH7 devices on the other hand employ the concept of dormant sessions or low power wake up for downlink transmissions. The heterogeneity of these RATs therefore requires a technology specific approach.

**Figure 1:**
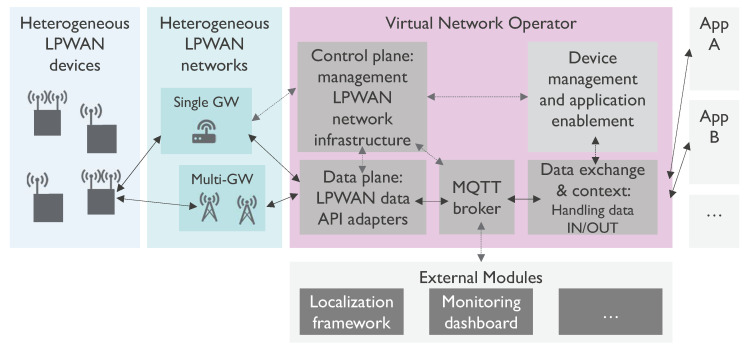
The modular implementation of the Virtual Network Operator allows easy addition of technologies and external modules. For example, a localization framework can extract data from the VNO’s MQTT broker and respond with a location estimate via the same path.

Once deployed, the network infrastructure will have to communicate with the corresponding adapter. Since every technology uses a different data format, the adapters are required to convert the data to a unified format and pass it to the applications on the right-hand side (and vice versa). Figure 1 displays a high level overview of the proposed architecture. This figure also illustrates that the VNO can interact with external modules such as a localization framework or a monitoring dashboard through the use of a Message Queuing Telemetry Transport (MQTT) broker.

In order to exchange data between two endpoints (i.e., the LPWAN device and the application) over multiple communication technologies, a different payload encoding for every technology should be avoided. As the internet has been dominated by TCP-UDP/IP protocols for the past 40 years, a CoAP/UDP/IPv6 based approach has been put forward, which enables a large plane for interoperability. In addition, the portability, where different applications can be used independent of the underlying protocols, provide a major benefit of this protocol stack. However, due to the restricted payload size of the LPWAN technologies, the overhead of CoAP/UDP/IPv6 makes this approach infeasible. As a result, a novel compression technique, named Static Context Header Compression (SCHC), has been used to compress the protocol headers by up to 95% [18].

### 3.2. Multimodal Localization

Our multimodal localization framework is designed in such a way that it can be implemented in two different ways. First, it can be added to a VNO as a modular component. For example, the VNO that was described in Section 3.1 collects messages from multiple LPWANs, and forwards them to the localization component to obtain a location estimate. Second, the framework can be used as a stand-alone service which is fed by multiple input sources, i.e., LPWAN network operators.

**Figure 2:**
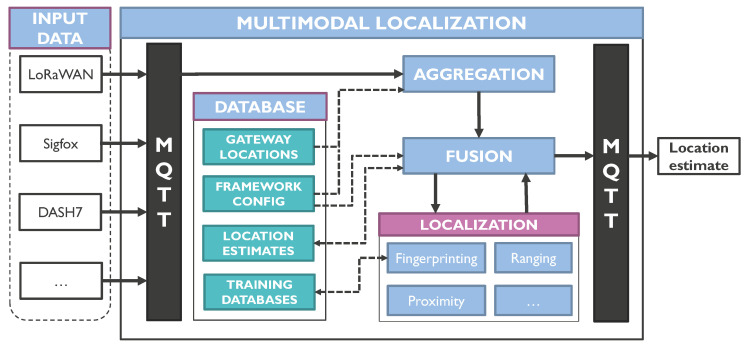
This figure provides a concise overview of our multimodal localization framework, which can be used together with our VNO.

Figure 2 illustrates the architecture of the framework. Here, we see that input data from a VNO or from other network operators is published to the framework through the MQTT protocol. Depending on the configuration of the framework, which is stored in the framework’s local database, the aggregation module subscribes to certain input sources. This can be a private or public LPWAN operator, or the MQTT broker of the VNO that is proposed in Section 3.1. Metadata from these sources are compared to the local database to extract the locations of receiving gateways if possible. This way, the aggregation module accumulates all relevant information such as RSS, timing, AoA, etc. which is required for localization. However, each input source can have a different metadata structure and content. For example, a LoRaWAN message from a private network operator contains all necessary information of all receiving gateways in a single Extensible Markup Language (XML)-formatted message, whereas a public Sigfox operator forwards a separate JavaScript Object Notation (JSON)-formatted message for each receiving gateway. Therefore, the aggregation module has the important task to store all information in a generic message format before forwarding it to fusion module. If a new input source must be added to the framework, it suffices to add a parser to the configuration database to translate the new input data to the framework’s generic format. On the other hand, this translation is not required if the aggregation module listens to the MQTT broker in the VNO, as depicted in Figure 1. In this case, the VNO translates its input to a generic format that can be shared with the localization framework.

**Figure 3:**
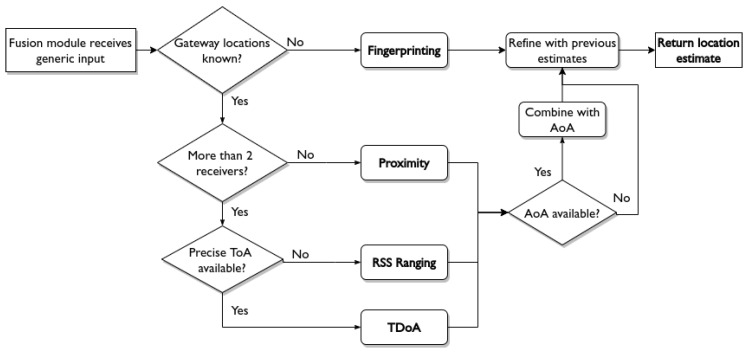
An example on how the fusion module can decide which localization method to use, based on the available input and the configuration that was set up by the user.

Next, the fusion module decides which method has to be applied to get an optimal location estimate. This decision can depend on elements such as the characteristics of the current wireless communication protocol or the input data from the aggregation module, i.e., the amount of receivers and their locations, the availability of RSS measurements, precise ToA timestamps and AoA estimates. Furthermore, the fusion module can take previous location estimates into account to refine new estimates. Figure 3 demonstrates how the fusion module can make a decision based on information that is made available by the aggregation module, and the configuration that is set up by the user. In this example, the first check determines whether the location of receiving gateways are known. If this is not the case, the fusion module will use a fingerprinting training set from the framework’s database to locate the multi-RAT IoT device. If the gateway locations are known, a choice is made between proximity, ranging, or TDoA localization depending on the number of receiving gateways and the availability of precise ToA timestamps from those gateways. When the module decides upon a localization method, that method is applied to the LPWAN message using parameters that can be tuned by the user in the framework configuration. The next step verifies if the input data contain AoA measurements. If so, the estimate that was calculated with the chosen localization method can be combined with AoA as described in [14]. Finally, previous location estimates for a device in the local database can refine the new location estimate, e.g., via Bayesian filtering [19].

The fusion module can be configured to meet specific constraints and requirements of an IoT application. For instance, choosing a propagation model that best matches the area of interest can lead to better results with RSS ranging. In addition, the user can tune fingerprinting hyperparameters, choose between different TDoA solvers, select which type of filtering should be used to refine location estimates, etc. The fusion module is not limited to the example in Figure 3, as the decision workflow can be extended if other types of input data are made available.

The final result is published on an MQTT broker where other services such as the VNO can obtain the location estimate of the transmitting LPWAN device. Furthermore, this location estimate can be utilized by the VNO for location-based handovers across LPWANS, as described in Section 3.1. To further clarify the workflow that was outlined in this section, we provide implementation examples of our framework in Section 4.

### 3.3. Location Estimation Reliability

Location-based services in their practical deployments generally feature certain inaccuracies. These inaccuracies, which are primarily characterized by the amplitude of estimation errors, should in many scenarios be leveraged jointly with location estimates to indicate how reliable they are. However, conjunct usage of locations and their corresponding errors is currently rarely the case, as discussed in [20]. The main reason for that lies in the fact that current approaches for estimating these errors predominantly rely on static performance benchmarks that are carried out immediately after deploying a localization solution [21]. These benchmarks typically provide some aggregated statistical metric (e.g., average value) for characterizing the estimation errors of the solution for the whole deployment environment [22]. Such spatially aggregated metrics do not account for the fact that errors usually substantially fluctuate along both temporal and spatial dimensions. Example-wise, it has been demonstrated that the inaccuracy for a variety of localization solutions are significantly larger at the edges of an environment, compared to its center [23]. In summary, location estimates are currently not used together with the corresponding estimates of localization inaccuracy because the aggregate statistical metrics cannot accurately characterize individual estimation errors.

To address this issue, we propose three procedures for estimating the reliability of a location estimate for RSS-based fingerprinting, which is one of the most promising localization solutions for indoor and GPS-denied outdoor environments. Although only one of these procedures is currently added to our localization framework, we intend to implement two more advanced Artificial Neural Network (ANN)-based algorithms in our future work. Therefore, we believe that these algorithms are worth mentioning here as well.

In our first basic approach, we explore a correlation between the number of receiving gateways and the calculated estimation error of a localization method. For this purpose, we use a large ground-truth dataset with Sigfox messages that was collected in our previous work [24]. We group the messages based on the number of gateways by which they were received and calculate a fingerprinting location estimate for each message in every group. As the GPS locations of all messages in the dataset are known, the actual estimation error of all location estimates can be calculated and, consequently, we are able to determine the 95th percentile error of each group. This number is used as the estimated reliability for the number of receiving gateways that is linked to that group, e.g., when a fingerprinting estimate is calculated for a Sigfox message that is received by six gateways, the reliability of that estimate equals the 95th percentile error of the fingerprinting estimates of all messages that were received by six gateways. The same procedure can be repeated for other localization algorithms such as RSS ranging if the gateway locations are known. This would allow the framework to make a substantiated choice on an optimal algorithm, based on the number of receiving gateways. Of course, a large ground-truth LPWAN dataset would have to be created for every area of interest to implement this procedure for reliability estimation.

Our two remaining procedures utilize the low-level signal features used for generating location estimates, as well as off-the-shelf regression and ANN algorithms. Specifically, we train the procedures with RSS values collected from different gateways used for fingerprinting at various locations in an environment of interest, as well as with the observed inaccuracy in case an estimate is calculated using these RSS values. Using the trained models, we are able to estimate the localization errors at new locations based solely on the observed RSS values at these locations. We also consider the usage of the location estimate as an optional additional input feature for the algorithms. We experimentally evaluate the optimally parameterized methods for LoRaWAN and Sigfox-based outdoor fingerprinting in several evaluation scenarios. Our results show that the proposed procedures are indeed feasible for estimating individual errors in fingerprinting, as they significantly and consistently outperform the baseline based on static performance benchmarks. Moreover, we show that the ANN-based method outperforms the regression-based ones across different environments and fingerprinting technologies. This is because, in contrast to regression, ANNs has a substantially larger number of tunable hyperparameters, which enables their optimization and fine-grained tuning for the problem at hand. More details on the proposed methods and their performance results can be found in our previous work [25,26].

## 4. Application Examples

This section demonstrates the benefit of implementing a multimodal localization framework in IoT applications. We discuss two use case examples and illustrate the expected location accuracy that can be achieved based on related work on LPWAN localization from Section 2.2. The first example combines GNSS, Sigfox, and DASH7 to keep track of valuable museum artworks. The second example is a GNSS-less tracking and monitoring application for construction equipment. In our future work, we intend to validate our framework by implementing it in similar IoT applications.

### 4.1. Use Case 1: Tracking Valuable Museum Artworks

In the first use case, valuable works of art are tracked while they are transported between a storage facility and a museum. Of course, tracking such costly assets requires a higher location accuracy than what can be achieved with localization via public LPWANs. Therefore, classic GNSS solutions have to be implemented in this use case. Before leaving the storage facility, a multi-RAT IoT device provided with a GPS receiver is mounted to the artwork’s container. The device can communicate through a public Sigfox network as well as a private DASH7 network at the museum. Hence, the aggregation module in the localization framework subscribes to two independent input sources.

**Figure 4:**
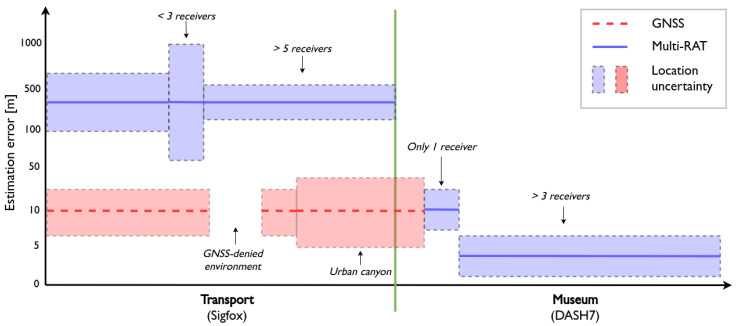
The expected accuracy and reliability for both GNSS as GNSS-less localization with our multi-RAT artwork tracking device. The expected estimation errors are based on results from related work in Section 2, and are displayed on a logarithmic axis.

Every ten minutes, the current GPS coordinates of the device are transmitted via a Sigfox message. As illustrated in Figure 4, urban canyons can affect the reliability of a GPS receivers because the lack of Line of Sight (LoS) between the receiver and GPS satellites increases the uncertainty of the location estimate. In GNSS-denied environments such as tunnels or indoor areas, it is not even possible to obtain a location estimate through GPS. This would mean that the location of our precious artworks would be unknown at that time. Thus, our localization framework is used as a fallback to ensure a location estimate at all times. As we know both the storage facility and the museum lie within an urban area of which we have a Sigfox fingerprinting database, the fusion module is configured to apply fingerprinting localization if a Sigfox message is received. Related work that used the same Sigfox fingerprinting database reports a mean estimation error of 319 m [7]. Hence, we can conclude that it is feasible to achieve a similar result for our artwork use case. Although this error has a significant magnitude, it surpasses not having an estimate at all in GNSS-denied environments. Additionally, the reliability of the resulting location estimate is calculated based on the number of receiving Sigfox gateways, as described in Section 3.3. This allows us to determine the uncertainty of a location estimate. Figure 4 illustrates that this uncertainty increases if less Sigfox gateways are available. Specifically, the location uncertainty with fingerprinting localization ranges from 650 m to 1000 m depending on the amount of receivers. In our future work, we will investigate other ways to calculate location uncertainty by integrating the ANN-based procedures of Section 3.3 in our framework.

Meanwhile, the tracking device periodically attempts to connect with the museums’ DASH7 network, with a fixed interval of one minute. This interval could also be changed dynamically by applying the location-based handover mechanism that is described in Section 3.1. Upon arrival at the museum, a DASH7 gateway that is installed there receives these transmissions and forwards them to the VNO. Figure 4 shows what happens when the VNO forwards this information to the localization framework. Initially, a GPS location is still available because DASH7 messages are received while the device has not yet entered the museum. However, GPS cannot be used anymore when the device enters the museum and the localization framework has to switch to other methods to calculate the artworks’ location. In the museum lobby, signals are received by a single DASH7 gateway, which causes the framework to choose for proximity localization and refining the location estimate with recent GPS data. As soon as more than three gateways start receiving transmissions, the framework can switch to multi-wall model localization and achieve a mean estimation error of 1.27 m [9].

Concisely, this example demonstrates that multimodal localization can optimize location accuracy and reliability depending on the environment and the constraints of the active LPWAN.

### 4.2. Use Case 2: Construction Tool Monitoring and Tracking

**Figure 5:**
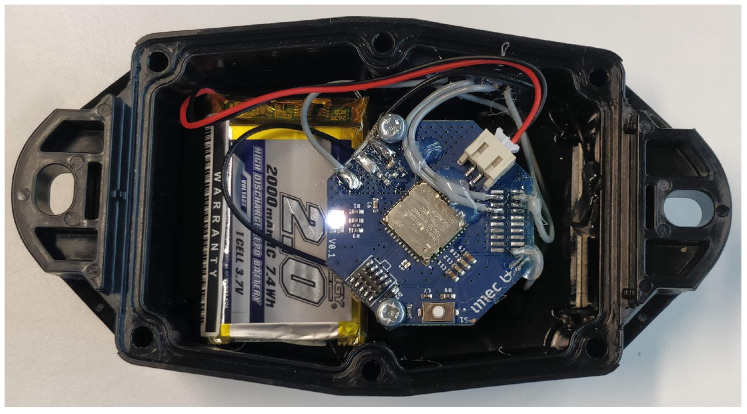
Our multi-RAT LPWAN device is able to switch between Sigfox, LoRaWAN, and DASH7 communication.

Damage costs are very high when excavator tools are not maintained properly. In the second use case, we collaborate with a construction company to develop a solution for monitoring the usage of these tools, and alerting the company if a certain usage threshold is exceeded. In order to optimize their work schedules, the company also wants to know on which construction site their tools are located. In this part of the use case, we need to classify roughly where the tool is located, so GPS-like accuracy is not required. Thus, we developed a GPS-less multi-RAT device prototype that can be attached to an excavator tool, with an accelerometer that was added to detect motion and count how frequently a tool is being used. Although the localization framework does not depend on this additional hardware, accelerometer data could optionally be used as context information to refine a location estimate. The multi-RAT LPWAN device can be seen in Figure 5.

Contrary to the first use case, the device is able to switch between DASH7, LoRaWAN and Sigfox communication. Because of the limited amount of memory that is available on the hardware, only the DASH7 and LoRaWAN protocol stacks could be implemented on the primary micro-controller, an external module had to be attached to facilitate Sigfox communication. Nonetheless, the switching between all three protocols was fully arranged via the primary micro-controller. In order to perform this switch as efficiently as possible, we apply the vertical hand-off algorithm that is described in Section 3.1. The algorithm takes the following considerations into account:

1. When connected to a network, the device should confirm at regular intervals whether it is still connected to this network
2. As some technologies are more powerful than others, a distinction should be made between these technologies

For our use case, this means that an acknowledgement has to be requested by the device after every transmission, regardless of its active communication protocol. When an acknowledgement is not received for multiple transmissions, the device has to switch to a different network because the current network is probably unavailable. On the other hand, the device should periodically poll for a more powerful network, even if the current network is still available. Hence, a hierarchy of networks has to be created so that the device can decide if it can switch to a more powerful network. In our use case, DASH7 is on top of the list as multiple gateways are installed at the company headquarters. The next best choice is LoRaWAN because a private LoRaWAN network is set up with a single gateway at one of the construction sites. If neither DASH7 nor LoRaWAN is available, a public Sigfox network with nationwide coverage is used as a last resort. As described in Section 3.1, a VNO is used to interface with all three IoT networks, with our localization framework implemented as a modular component [15]. In this implementation, the aggregation module in the localization framework can adopt the generic message format that is used by the VNO because this format holds all the information that is needed to apply localization methods. Its only task is to read incoming messages and attempt to retrieve the locations of the receiving gateways from the local database.

**Figure 6:**
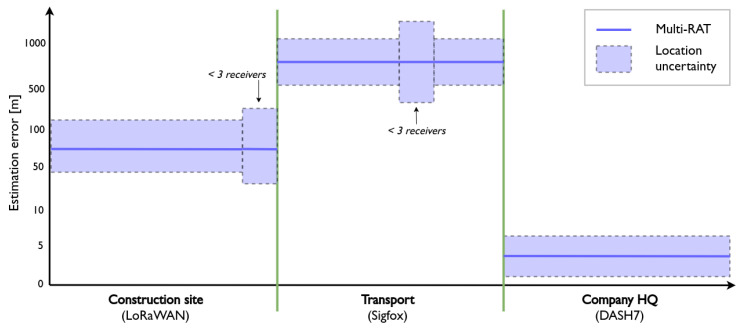
The expected location accuracy and reliability of the construction tool use case. GNSS is omitted from this figure, as the tracker does not contain a GNSS receiver. The expected estimation errors are based on results from related work in Section 2, and are displayed on a logarithmic axis.

Figure 6 gives an idea of the expected location estimation accuracy in this use case. An excavator tool that is in use on a construction site triggers the motion detection algorithm on the tracking device, which increases a usage time counter based on the duration of the detected motion. Whether or not the usage time has been updated, it is transmitted via one of the three networks once every ten minutes, initially over DASH7. As the DASH7 network is not available on the construction site, the device receives no acknowledgements and switches to the next best network (LoRaWAN) after four attempts. Now, the VNO reports to the localization framework that a LoRaWAN message was received one or more gateways that are located on the construction site. The estimation accuracy that can be achieved depends on the number of LoRaWAN gateways that are present on the construction site, and on the features that they support. The presence of two synchronized gateways and one AoA-enabled gateway allows for a combination of TDoA and AoA localization, which can lead to a mean error of 122 m [14]. As our device contains an accelerometer, sensor data can also be taken into account to refine the location estimates. Related research on this topic reports a median error of 75 m when taking sensor data into account [11]. As a result, the location framework can classify on which construction site the tool is located, and reports the site location back to the VNO. This satisfies the basic requirement of this part of the use case.

Next, the excavator tool has to be transported back to the company headquarters, as the usage counter indicates that maintenance is required. During transport, the LoRaWAN network will become unavailable which causes the device to attempt a switch to DASH7. Specifically, there is a new attempt to connect to the DASH7 network after every four LoRaWAN messages. Because the device is not in the range of neither the DASH7 nor LoRaWAN networks, it will ultimately switch to Sigfox communication. In a worst-case scenario, the network switch to Sigfox can have a delay of up to 40 min, as there is an interval of 10 min between each message. The location-based handover mechanism that is explained in Section 3.1 could significantly decrease this delay by dynamically changing the interval between consecutive transmissions.

Because there is a distance of tens of kilometers between the construction site and the company headquarters, building a fingerprinting database for this area is nearly impossible. Thus, the fusion module applies RSS ranging if a Sigfox message is received, resulting in a location error of hundreds of meters depending on the number of receiving gateways, the characteristics of their environment and their distance to the transmitter. If less than three Sigfox gateways received the transmission, proximity localization is applied using the location of the gateway with the strongest RSS as the location estimate. During this phase of the use case, the estimation accuracy can range from 722 m up to more than 1000 m [8].

Finally, the device switches to DASH7 communication shortly after arriving at the headquarters because it continues to poll for a more powerful IoT network. Similar to the museum example that we described in Section 4.1, the localization framework switches accurate localization with a multi-wall propagation model. With a mean error of 1.27 m, the DASH7 network at the company headquarters supports a warehouse management solution that accurately reports the location of excavator tools.

This industrial IoT use case illustrates the benefit of applying multi-RAT communication in conjunction with a multimodal localization framework. A similar configuration of the multimodal localization framework can be readily applied to other use cases such as smart ports, where there is an emerging need for consistent tracking and tracing of heterogeneous goods.

## 5. Conclusions

Location-based services for IoT applications face many challenges that depend on the requirements of the application. Unfortunately, there is no single solution that can deal with all of these challenges, which is why a combination of multiple solutions is required. In this paper, we propose a multimodal localization framework that can be integrated with a VNO. This framework enables reliable localization for multi-RAT IoT devices that transition between indoor and outdoor environments, which significantly increase the flexibility of the IoT applications that adopt this concept. The benefits of our framework design are demonstrated through two IoT use case examples that involve asset tracking in multiple environments, using a device with a single sub-GHz radio chip that can switch between Sigfox, LoRaWAN, and DASH7. In these specific examples, the achievable location accuracy with these LPWANs can range from 1 m up to more than 1000 m, depending on the constraints of the environment and the devices’ active LPWAN. As we have seen in related work in Section 2, such high errors are inherent to localization with public LPWANs in large public areas. Our future work includes implementing the framework in real use cases with controlled private LPWANS to validate the feasibility of achieving lower estimation errors in such scenarios. For example, the area of interest of a smart port could be covered by a private LoRaWAN network with TDoA and AoA-enabled gateways for outdoor localization, resulting in a mean error of 122 m instead of 1000 m [14]. Similar to the construction equipment example in Section 4.2, accurate indoor localization for warehouse management can be set up with a DASH7 network. Concisely, the expected accuracy with LPWAN localization depends on the constraints of the available LPWANs and the size of the area of interest. Our framework serves as a means to enable persistent location-based services for multi-RAT IoT devices that communicate through those LPWANS. Moreover, the location estimate that is obtained with our framework can be used for location-based handover mechanisms, allowing a multi-RAT device to optimize its energy consumption.

In our future work, we intend to improve our framework in multiple ways. First, we want to extend our work to include not only sub-GHz LPWANs, but also 2.4 GHz and Ultra Wideband (UWB) networks. This would allow us to achieve highly accurate localization for indoor applications such as warehouse management. Of course, this also means that our multimodal transmitter of Section 4.2 has to be provided with additional communication hardware to connect to these networks. Second, we will introduce sensor data to the localization framework. For instance, the multimodal device that is shown in Section 4.2 contains an accelerometer to detect the motion of excavator tools. Data from this accelerometer could also be used for dead reckoning localization, or as context information to improve the accuracy of a location estimate. Lastly, we aim to enhance the estimated reliability of our localization methods by adopting the procedures which are proposed in Section 3.3. After integrating these features, we intend to validate our framework design by implementing it in a real IoT application in combination with a VNO.
